# Solvent Removal Salicylic Acid-Loaded Myristic Acid-Based In Situ Forming Gel

**DOI:** 10.3390/gels12030220

**Published:** 2026-03-06

**Authors:** Kritamorn Jitrangsri, Napaphol Puyathorn, Sai Myo Thu Rein, Jitnapa Sirirak, Parichat Chomto, Thawatchai Phaechamud

**Affiliations:** 1 Department of Industrial Pharmacy, School of Pharmacy, Walailak University, Nakhon Srithammarat 80160, Thailand; kritamorn.ji@wu.ac.th; 2 Department of Pharmaceutical Sciences, Faculty of Pharmacy, Chiang Mai University, Chiang Mai 50200, Thailand; napaphol.p@cmu.ac.th; 3 Department of Pharmacognosy, University of Pharmacy, Mandalay 05012, Myanmar; saimyothurein@gmail.com; 4 Department of Chemistry, Faculty of Science, Silpakorn University, Nakhon Pathom 73000, Thailand; sirirak_j@silpakorn.edu; 5 Division of Industrial Pharmacy, Faculty of Pharmacy, Silpakorn University, Nakhon Pathom 73000, Thailand; chomto_p@su.ac.th

**Keywords:** salicylic acid, in situ forming gel, myristic acid, solvent removal

## Abstract

This study aimed to develop a solvent removal-based in situ forming gel (ISG) loaded with salicylic acid (SAL) using myristic acid (MYR) as a matrix-forming agent. SAL-loaded MYR-based ISGs were prepared using N-methyl-2-pyrrolidone (NMP) or dimethyl sulfoxide (DMSO) as solvents and evaluated for physicochemical properties, matrix formation behavior, mechanical characteristics, and in vitro drug release. Increasing MYR content influenced viscosity, gel formation kinetics, and depot integrity, resulting in prolonged SAL release of up to 20 days in DMSO-based formulations. The release kinetics were best described by the Peppas–Sahlin model, indicating diffusion-dominated drug transport. The selected formulation containing 30% *w*/*w* SAL and 20% *w*/*w* MYR exhibited acceptable injectability, reproducible in situ matrix formation, and sustained drug retention. Antimicrobial testing confirmed that SAL retained biological activity against oral pathogens following incorporation into the ISG system, although solvent contributions to antimicrobial effects were also observed. These findings demonstrate the feasibility of a MYR-based ISG system in which SAL contributes to both therapeutic activity and matrix formation, supporting its potential for localized oral drug delivery.

## 1. Introduction

Salicylic acid (SAL), also known as 2-hydroxybenzoic acid, is a naturally occurring compound that has been isolated from the bark of willow trees (*Salix* spp.) [1]. In pharmaceutical and cosmetic industries, SAL is widely recognized and utilized due to its diverse bioactivities, including keratolytic, anti-inflammatory, and antimicrobial properties [2,3,4]. These properties have made SAL a common therapeutic agent in the treatment of various skin diseases such as acne, psoriasis, warts, and superficial mycosis [5,6,7,8]. One of the primary limitations of SAL in these applications is its poor water solubility [9] which significantly reduce its efficacy by leading to suboptimal therapeutic outcomes due to inadequate bioavailability and penetration through biological membranes [10]. Although SAL has low water solubility, it is interesting study as a matrix-forming agent or active compound in in situ forming gel (ISG).

Myristic acid (MYR), a long-chain fatty acid derived from natural sources, is used as a multipurpose food additive and flavor adjuvant [11]. Compared with other fatty acids and biodegradable polymers commonly investigated as matrix formers, MYR offers a favorable balance of physicochemical properties for in situ forming gel (ISG) systems. Unlike shorter-chain fatty acids, which exhibit higher aqueous solubility and may result in rapid matrix erosion, MYR is very slightly soluble in water while remaining highly soluble in organic solvents [12]. It has favorable characteristics for matrix formation in in situ forming gels (ISGs) by showing excellent solubility in organic solvents while being very slightly soluble in water [13,14]. This property allows for the possibility of easy gelation upon contact with physiological fluids, making it a potential candidate for the development of ISGs. MYR has been previously used in various pharmaceutical applications, such as cream base for delivery of clobetasol propionate [15] or as a matrix former of the implant for sustained delivery of lidocaine [16], or as an emulsifying agent of the microemulsion for curcumin topical formulation [17]. Compared to previously reported fatty acid–based ISGs employing stearic acid or lauric acid, the combination of MYR and SAL offers distinct advantages. MYR provides sufficient hydrophobicity to retard slow exchange while maintaining acceptable injectability, whereas SAL contributes both therapeutic activity and structural reinforcement of the matrix. This synergistic interaction might enable prolonged drug retention without excessive viscosity or injection force, addressing limitations observed in earlier fatty acid ISG systems.

In situ forming gels (ISGs) have emerged as a promising drug delivery system for localized and sustained release of therapeutic agents [18]. These systems undergo a transition from a liquid to a gel-like or solid state upon administration, enabling controlled drug release at the application site [19,20]. ISGs typically consist of three main components: an active compound, a matrix-forming agent (commonly a biodegradable polymer or lipid) that is soluble in a water-miscible organic solvent but insoluble in water, and a biocompatible organic solvent [21]. ISGs can be formed through several mechanisms, including phase separation, covalent cross-linking, and solvent removal–induced gelation. For example, progesterone dissolved in ethanol with medium-chain triglycerides forms an ISG with a dense structure and controlled release behavior upon intramuscular injection, where gel formation occurs via phase separation [22]. Covalent cross-linking–based ISGs involve gelation through radical formation induced by chemical reactions [23], temperature changes [24], pH adjustments [25], or UV exposure [26].

Among these approaches, solvent removal-induced ISGs are particularly attractive due to their simple gelation mechanism and favorable safety profile. Upon contact with physiological fluids, a bidirectional solvent exchange occurs, whereby the water-miscible organic solvent diffuses outward while water penetrates inward. This process reduces the solubility of the matrix-forming agent in the formulation microenvironment, triggering its precipitation and self-assembly into a gel or solid matrix. Because gelation is driven solely by solvent diffusion and phase inversion, this system avoids the use of potentially harmful chemical initiators, cross-linking agents, or external stimuli such as heat, UV irradiation, or pH changes, thereby reducing cytotoxicity and simplifying formulation requirements. In addition, solvent removal-induced ISGs allow minimally invasive administration with in situ solidification, resulting in safer and more biocompatible drug delivery systems. The permeability behavior of salicylic acid-loaded fatty acid ISGs has previously been investigated using molecular dynamics simulations and confocal imaging techniques [27]. The selection of an appropriate matrix-forming agent is therefore critical for the successful development of solvent removal-induced ISG formulations. In this study, salicylic acid (SAL) and myristic acid (MYR) were selected as the active compound and matrix-forming agent, respectively, due to their favorable pharmacological activity and physicochemical compatibility with the solvent exchange-driven gelation mechanism.

Physicochemical parameters such as density and surface tension play an important role in the performance of ISG formulations intended for oral administration [28,29]. Density influences the sedimentation behavior of the formulation and the ease of administration. Surface tension governs the wetting behavior and spreading of the formulation on the oral mucosal surface, which in turn affects the extent and uniformity of solvent exchange with biological fluids. Efficient wetting and spreading facilitate rapid solvent diffusion and consistent in situ gel formation, thereby contributing to reproducible drug release profiles. Consequently, characterization of density and surface tension provides clinically relevant insight into formulation handling, administration, and in vivo gelation behavior.

The primary objective of this study was to develop and characterize a solvent removal-based MYR–SAL in situ forming gel system for localized oral drug delivery, in which salicylic acid functions as both a therapeutic agent and a structural contributor. The specific sub-objectives were to (i) investigate the influence of SAL and MYR concentrations on physicochemical, mechanical, and gelation properties; (ii) evaluate in situ matrix formation and sustained drug release behavior; and (iii) assess whether SAL retains antimicrobial activity against oral pathogens following incorporation into the ISG system.

Solvent removal-based in situ forming gel systems using fatty acids have been previously reported, yet most research treats the matrix former as the only structural element while the drug remains a passive payload. The present work uses salicylic acid at high concentrations, so it acts both as an active pharmaceutical ingredient and as a co-matrix-forming component. The dual function enables salicylic acid to affect phase inversion behavior, matrix crystallinity, and depot integrity, which supports changes in mechanical performance and sustained release. To our knowledge, no prior study has systematically examined the structural and therapeutic roles of salicylic acid within a fatty acid-based solvent removal ISG system designed for localized oral delivery. The formulation developed with salicylic acid and myristic acid advances solvent removal based ISG design for topical use in the oral cavity and supports targeted management of oral cavity disease. The results may indicate potential value for treating mouth ulceration by offering a more effective and more convenient option for patients.

## 2. Results and Discussion

### 2.1. Physicochemical Properties

#### 2.1.1. Appearance and Preparation

Salicylic acid (SAL) (10–60% *w*/*w*) and myristic acid (MYR) (20–30% *w*/*w*) combined with 30% *w*/*w* SAL were dissolved in various solvents including N-methyl pyrrolidone (NMP), dimethyl sulfoxide (DMSO), or 2-pyrrolidone (PYR), as outlined in Table 1 using a magnetic stirrer at room temperature until clear in situ forming gel solutions were obtained. The appearance of the formulation was assessed through visual inspection. Dissolution became incomplete when SAL exceeded 60 percent *w*/*w* or MYR exceeded 30 percent *w*/*w*, and excess solute was precipitated. In PYR, SAL above 40 percent *w*/*w* also failed to dissolve fully and led to precipitation of the surplus drug. All formulations prepared within the soluble ranges remained colorless and visually clear and showed viscosity suitable for handling.

#### 2.1.2. Density and Viscosity

Formulations containing different SAL concentrations in NMP, DMSO, and PYR were evaluated for appearance and physicochemical properties, with the results summarized in Table 2. Density increased in proportion to SAL concentration across all solvents. The rise in density is consistent with adding a higher molecular mass solute to lower molecular mass solvents, which increases mass per unit volume in the SAL loaded ISG. Viscosity also increased as SAL content increased in each solvent system. Similarly, the increase in viscosity reflects greater solute loading and stronger physical interactions among components, which raises resistance to flow.

With SAL content held constant, higher myristic acid (MYR) levels reduced formulation density in the DMSO system. The pattern is consistent with MYR having a lower density of 1.03 g/cm^3^ than DMSO at 1.1 g/cm^3^ [30,31]. Adding more MYR, hence, increased volume relative to mass and lowered the overall density. Density and viscosity also determine injectability and remain critical for accurate delivery of the formulation to the affected site.

#### 2.1.3. Surface Tension and Contact Angle

Surface tension and contact angle values are shown in Table 2. Surface tension describes the force at the boundary between immiscible phases, most often a liquid and air, and reflects cohesive forces among molecules at the liquid surface [32,33]. Higher SAL concentrations produced a slight rise in surface tension in NMP and DMSO, whereas PYR showed a marked increase as SAL increased. Stronger hydrogen bonding and electrostatic interactions involving SAL likely raised cohesive forces and increased surface tension in the ISG formulations [34]. This suggests that PYR may not be the most suitable solvent due to its lower spreadability on tissue surfaces. The high surface tension in PYR indicates poor spreading on tissue surfaces and less favorable mucosal wetting. PYR-based systems also displayed weaker gel formation behavior and less consistent sustained release than DMSO and NMP formulations, which further limits their overall suitability for localized oral delivery.

The contact angle is another important measurement that provides insights into the spreadability of a formulation. A lower contact angle reflects better wetting and spreading, whereas a higher contact angle indicates poorer wetting [35,36]. Contact angle increased in proportion to SAL concentration across all solvents on both glass and agarose gel surfaces, as depicted in Table 2. The higher values on agarose gel likely result from in situ gel formation upon exposure to phosphate buffer at pH 6.8 used in gel preparation, which promotes solid like matrix formation and raises the measured angle [37]. Contact angles for all formulations remained below 90 degrees, indicating acceptable wetting on the tested surfaces [38].

#### 2.1.4. In Situ Gel Transformation

**Figure 1:**
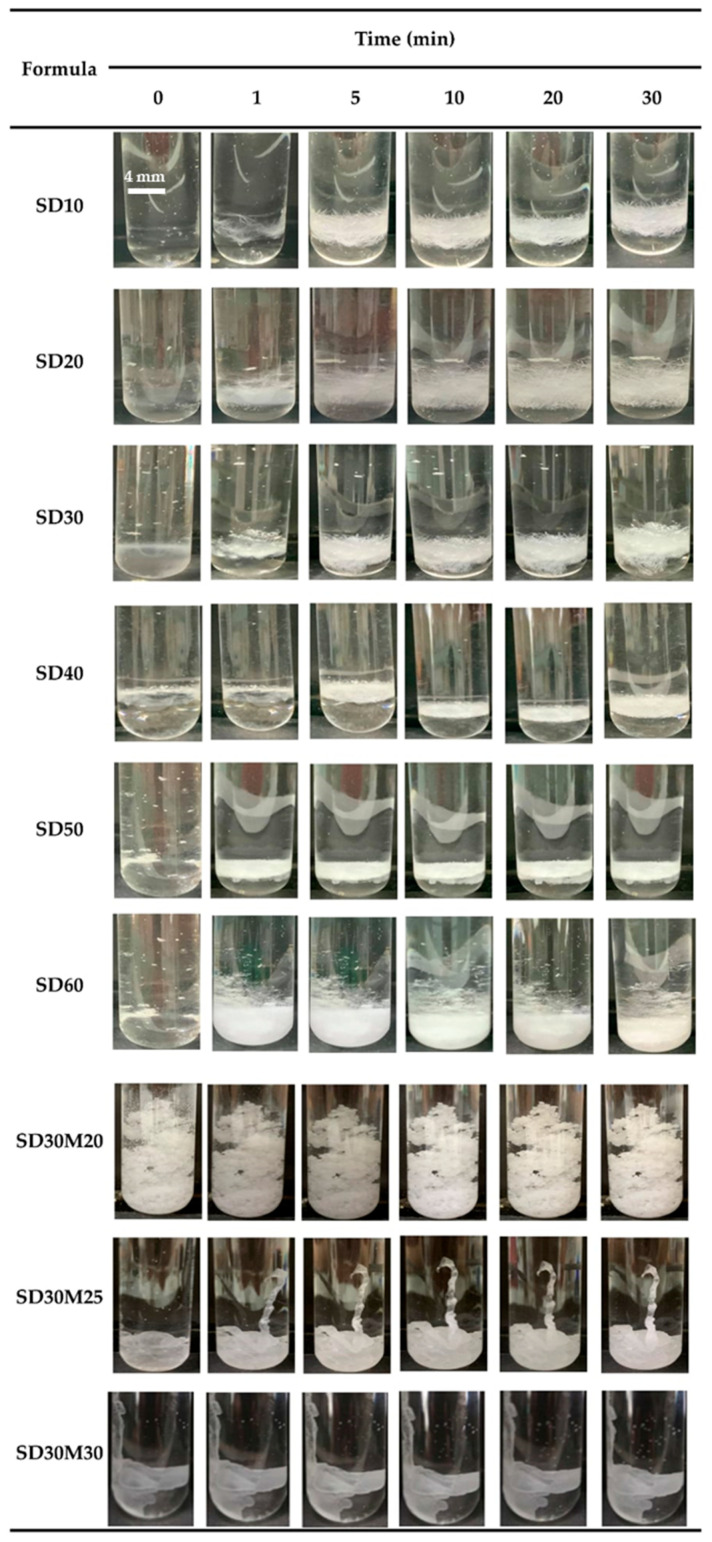
In vitro matrix formation behavior of SAL-loaded MYR-based ISG in DMSO after contact PBS pH 6.8.

The gel formation process of salicylic acid (SAL) at various concentrations (10–60% *w*/*w*) in NMP, DMSO, and PYR was examined by injecting the formulations into pH 6.8 phosphate-buffered saline (PBS). Photos were taken at different time intervals to monitor gel formation. The results demonstrated that gel formation was infrequent at SAL concentrations of 10–50% *w*/*w* in NMP, and only a slight gel formation was observed at 40% *w*/*w* SAL in PYR. In contrast, gel formation was easily achieved in DMSO, starting from a SAL concentration of 10% *w*/*w*. Moreover, as the SAL concentration increased, the gel formed more compact matrix and more rapidly over time, as seen in Figure 1.

**Figure 2:**
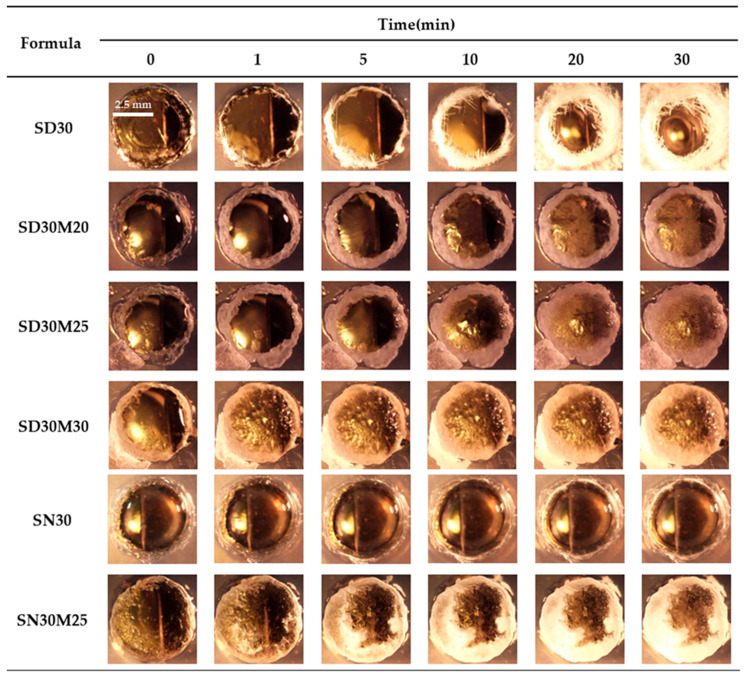
In vitro matrix formation behavior of SAL-based ISG and SAL-loaded MYR-based ISG in 0.6% agarose with PBS pH 6.8 (under stereomicroscope 24×).

Addition of myristic acid (MYR) enabled easier gel formation in both NMP and DMSO compared to formulations without MYR, and the gels formed immediately after injection and became denser over time [37,38,39,40,41]. Gel development was also examined in agarose gel wells under a stereomicroscope, as illustrated in Figure 2. Observations in agarose matched test tube results, with limited gel formation in NMP and more pronounced gel formation in DMSO at 30% *w*/*w* SAL. Gel formation appeared as a white solid advancing from the edge toward the center of the agarose over 30 min. MYR addition produced visible gel formation within the first minute and increased compactness over 30 min in both DMSO and NMP. Higher MYR levels produced earlier onset and a more compact matrix, supporting selection of NMP and DMSO as more suitable solvents than PYR.

In addition, higher MYR content appeared to speed visible gel or solid formation in injection and agarose gel models, yet the observation reflects early phase separation rather than full gel maturation [39]. MYR promotes hydrophobic interactions and early precipitation at the solvent water interface, which leads to faster formation of a visible solid matrix. Higher MYR levels also raise viscosity and hydrophobicity, which slows solvent water exchange and delays complete matrix consolidation and internal structural organization. Prolonged solvent removal permits MYR molecules to align and pack more efficiently, producing higher crystallinity in the final gel structure that matches SEM observations. Higher MYR content, hence, shortens the onset of visible gel formation while extending the maturation phase and yields a more crystalline matrix [40].

#### 2.1.5. Injectability Properties

Injectability refers to the ease with which a formulation can be administered through a syringe–needle system during injection [41]. Injectability is influenced by several factors, including formulation viscosity, flow behavior, and interaction with needle geometry. The acceptable injection force for injectable formulations is generally reported to be below 40 N, with values below 20 N considered more favorable for clinical handling [42].

In the present study, injectability was evaluated using a fixed needle gauge; therefore, differences in injection force primarily reflect variations in formulation viscosity under identical testing conditions. All ISG formulations prepared in DMSO or NMP exhibited injection forces below 3 N (Table 3), indicating easy administration to the target site. ISGs prepared in NMP required slightly higher injection forces than those prepared in DMSO, which is consistent with their comparatively higher viscosities [43].

Increasing MYR content resulted in a higher injection force and work of injection due to increased viscosity and density of the ISG solutions at higher MYR concentrations (Table 2). Nevertheless, the work of injection for all formulations remained below 50 N·mm, which is generally considered acceptable for injectable systems [44]. These results indicate that, despite variations in formulation composition, all ISGs demonstrated suitable injectability under the tested conditions.

#### 2.1.6. Mechanical Properties

The mechanical properties of the 3-day-equlibrium ISGs filled in 0.6% *w*/*w* agarose gel are shown in Table 3. The hardness of the formed ISG increased proportionally to the MYR content, as evidenced by the increased maximum force values. The ISGs prepared in DMSO without MYR addition had higher hardness than those prepared in NMP (SD30 vs. SN30). This is consistent with our previous study, which showed that the slower matrix formation process in NMP results in a more compact aged ISG at 3 days [28]. Additionally, the adhesion force was not significantly different between solvent types (SD30M25 vs. SN30M25), but it increased with increasing MYR content. This is likely due to the lipophilicity and waxy nature of MYR [11], which increases the adhesion of the ISG to the probe of the texture analyzer.

#### 2.1.7. In Vitro Drug Release

**Figure 3:**
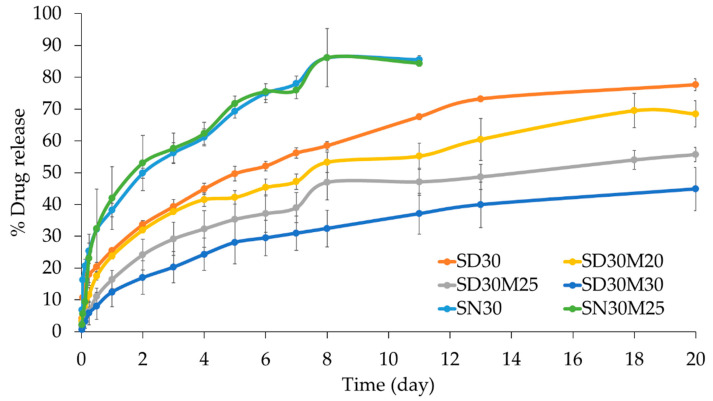
Comparison of release profile of SAL from SAL-loaded MYR-ISG system (n = 6).

The release profiles of SAL from the SAL-loaded ISG system, varying MYR concentrations, and solvent types (DMSO or NMP), are presented in Figure 3. Sink conditions were maintained throughout the release study by ensuring that the concentration of SAL in the release medium remained well below its saturation solubility. The control groups, SD30 and SN30, exhibited the most rapid SAL release. It is not surprising that in the absence of added MYR, the in-situ gel formed rapidly, as observed in Section 2.1.4, resulting in a swift release and inefficient retention of SAL. Comparing solvent types, as discussed in the matrix formation behavior above, ISG in NMP exhibited slower solidification than that in DMSO. Consequently, incomplete trapping of SAL in the matrix structure led to a faster release profile. In contrast, ISG in DMSO, with faster solidification and higher crystallinity in the ISG, demonstrated a better drug retention rate [45]. This can be attributed to the swift phase-inversion of DMSO, creating a denser depot structure and making it difficult for water to penetrate, thereby halting the solvent exchange process and consequently ceasing drug release until degradation initiates [46].

The inclusion of MYR decelerated matrix formation by delaying phase inversion development, as observed under stereoscope (Figure 2). It is noteworthy that the addition of hydrophobic compounds tends to slow down the matrix formation process, leading to prolonged sustained release of the active compound [21,47]. In this study, higher MYR content resulted in increased drug retention, extending up to a maximum of 20 days. This aligns with previous findings where the addition of MYR led to a slower release of etoposide, extending up to 30 days [48]. The ISG using MYR in this study demonstrated a longer sustained release of the active compound compared to previous studies using stearic acid or lauric acid, which could only maintain the release for about 6 days [18].

Although NMP and DMSO are high boiling point solvents, their removal in the present system does not occur via evaporation but through a solvent–water exchange mechanism. Upon contact with the aqueous environment, the water-miscible organic solvent gradually diffuses out of the formulation while water diffuses inward, triggering phase inversion and precipitation of salicylic acid and myristic acid to form a solid or semi-solid depot. Complete solvent removal was not directly quantified in this study; however, progressive solvent exchange was indirectly evidenced by rapid in situ matrix formation, sustained drug release behavior, and the solid remnants observed by SEM following dissolution testing. The final formed system is therefore a dense lipid-based depot rather than a conventional hydrogel, designed to retain the drug locally while allowing gradual solvent diffusion into the surrounding medium.

The release profiles of SAL were fitted to several kinetic models, including zero-order, first-order, Higuchi, Korsmeyer–Peppas, and Peppas–Sahlin equations. Model suitability was evaluated using multiple statistical criteria, namely the coefficient of determination (R^2^), Akaike Information Criterion (AIC), and Model Selection Criterion (MSC). As summarized in Table 4, the Peppas–Sahlin model consistently exhibited higher R^2^ values together with lower AIC and higher MSC values compared to the other models, indicating superior goodness of fit across all formulations [49]. The Peppas–Sahlin model describes drug release as a combination of Fickian diffusion (k_1_ term) and polymer relaxation or matrix reorganization (k_2_ term) [50]. In the present study, all formulations showed substantially higher k_1_ values than k_2_ values, with k_2_ being negligible or negative in several cases, indicating that relaxation-controlled mechanisms contributed minimally to SAL release [51]. Negative or near-zero k_2_ values have been reported in diffusion-dominated systems and are generally interpreted as reflecting a negligible contribution of relaxation or erosion processes rather than a physically meaningful negative mechanism. Furthermore, the diffusion exponent (m or n) values obtained from both the Korsmeyer–Peppas and Peppas–Sahlin models were consistently below 0.45, which is characteristic of Fickian diffusion–controlled release from non-swelling matrix systems [52]. This behavior is consistent with the solvent removal-induced gelation mechanism, where rapid phase inversion leads to the formation of a dense, diffusion-limiting lipid matrix. In the absence of MYR in NMP-based formulations (SN30), the release profile was better described by the Korsmeyer–Peppas model; however, the similar m and n values and the negligible relaxation contribution indicate that SAL release remained predominantly governed by molecular diffusion driven by the concentration gradient [53]. Although diffusion appears to be the dominant release mechanism based on kinetic modeling and structural observations, direct quantification of matrix erosion was not performed in this study; therefore, minor contributions from erosion cannot be fully excluded and warrant further investigation.

The in vitro release study was conducted in phosphate-buffered saline (PBS) at pH 6.8 and 37 °C to approximate the physiological conditions of the oral cavity, particularly saliva and gingival crevicular fluid, where pH values close to neutrality are commonly reported. These conditions were selected to simulate the immediate aqueous environment encountered by the injectable in situ forming gel following administration and to reproduce the solvent exchange-driven phase inversion mechanism that governs matrix formation and drug entrapment. Although the experimental setup does not fully replicate the complexity of in vivo, it provides a controlled and reproducible platform to evaluate matrix formation, matrix integrity, and diffusion-controlled drug release. The prolonged release of SAL observed for up to 20 days therefore reflects the ability of the MYR-based matrix to form a dense, diffusion-limiting depot upon aqueous exposure, which is a critical determinant of in vivo performance. Consequently, the in vitro dissolution model is considered physiologically relevant for comparative formulation screening, while further in vivo studies will be required to establish direct in vitro–in vivo correlation [54].

Moreover, the selection of the formulation containing 30% *w*/*w* SAL and 20% *w*/*w* MYR was guided by overall optimization of physicochemical, mechanical, and biological performance rather than release kinetics alone. SD30M20 demonstrated lower viscosity and lower injection force than formulations with higher MYR content of 25 to 30% *w*/*w*, while retaining adequate mechanical strength and depot integrity. Formulations with lower MYR content produced faster drug release and weaker retention. Antimicrobial activity against oral pathogens remained comparable across all SAL loaded MYR-based ISGs, so higher MYR content did not improve antibacterial efficacy. The 30% *w*/*w* SAL and 20% *w*/*w* MYR formulation therefore offered the best balance, with acceptable injectability, reproducible matrix formation, stable mechanical properties, effective antimicrobial activity, and prolonged sustained release. The formulation was selected as the optimal candidate because it provided the strongest overall compromise across the evaluated systems.

#### 2.1.8. Surface Topography

**Figure 4:**
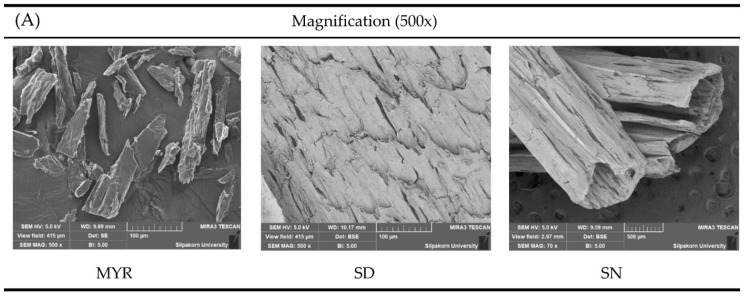

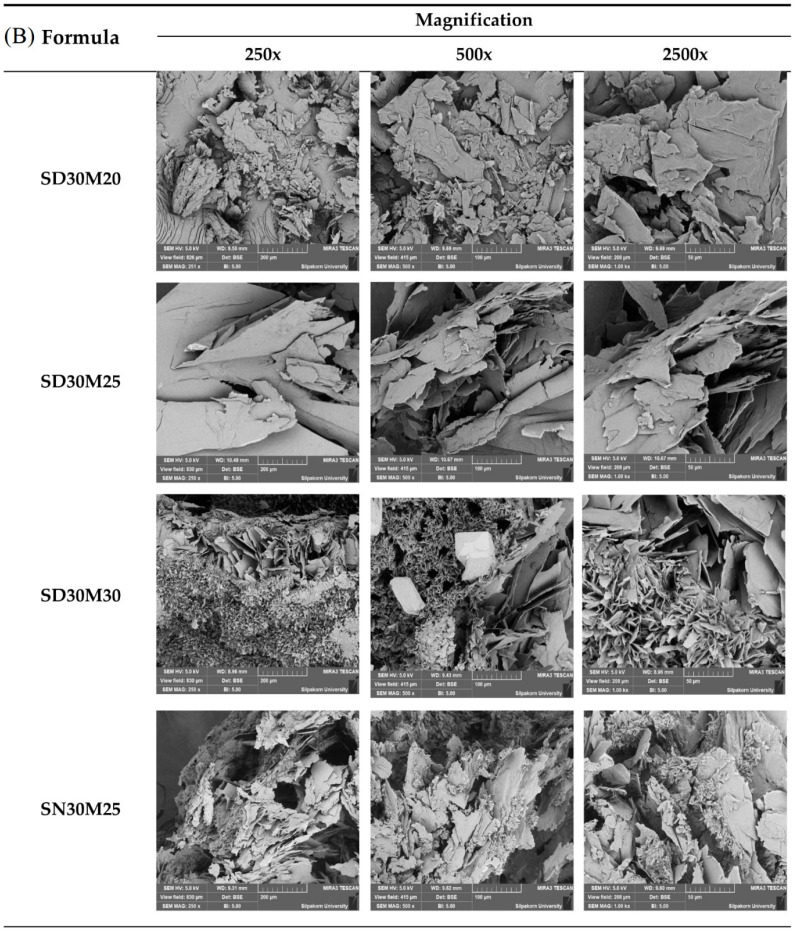
SEM photographs of MYR, SD and SN remnants (**A**) and SAL-loaded MYR ISG systems using DMSO and NMP as the solvents after drug release test in PBS pH 6.8 for 7 days (**B**).

The compact morphology observed in SEM is consistent with the higher crystallinity indicated by XRD patterns and the thermal behavior observed in DSC, which supports more structured matrix formation as shown in Figure 4. SEM images also distinguished the physical characteristics of intact MYR and SAL. Intact MYR displayed a flake-like powder structure, whereas SAL in both DMSO and NMP showed cracked surfaces and a hollow rod like morphology in Figure 4A.

At ×2500 magnification, dry remnants of SAL in DMSO formulations containing MYR showed crystalline flakes in Figure 4B. Larger flakes were attributed to MYR, whereas smaller and more finely dispersed flakes were attributed to SAL. The lack of small flakes on SD30M20 and SD30M25 surfaces suggests complete SAL release and agrees with DSC, TGA, and XRD results. Small flakes remained in SD30M30, indicating residual SAL retained within the MYR matrix. The pattern matches the release profiles, as higher MYR content corresponds with slower SAL release and greater SAL retention within the matrix. Similar observations have been reported, where MYR addition reduced the release rate of the active compound due to strong matrix-forming capacity and hydrophobic character [55].

Furthermore, comparison of DMSO and NMP solvents in SD30M25 and SN30M25 formulations demonstrated higher crystallinity in ISGs prepared with DMSO. Higher crystallinity corresponds to faster matrix formation in DMSO-based systems than in NMP based systems, as indicated by the appearance of small SAL flakes on the ISG surface. The observation is consistent with earlier findings reporting quicker solidification of ISG mass separated from DMSO than from NMP, which was associated with higher drug retention [45].

### 2.2. Bioactivities

Antimicrobial Activities

The cup agar diffusion method was utilized to assess the antimicrobial efficacy of the developed ISG formulations against various pathogenic strains, including *Staphylococcus aureus*, *Escherichia coli*, *Porphyromonas gingivalis*, *Candida albicans*, *Candida krusei*, *Candida lusitaniae*, and *Candida tropicalis*. The inhibition zone diameters are presented in Table 5. The selection of these pathogens was based on their relevance to oral cavity-related infections and their diverse spectrum of pathogenicity [56].

Remarkably, the negative controls (DMSO and NMP) exhibited antimicrobial activity (*p* < 0.05), occasionally displaying higher activity compared to the inclusion of the active compound SAL, particularly against Gram-positive pathogen *S. aureus*, Gram-negative pathogen *E. coli*, and fungal pathogens *C. albicans*, *C. krusei*, and *C. tropicalis* (*p* < 0.05). This can be attributed to the well-documented antimicrobial effects of these solvents, especially NMP, which is used as a solvent in the commercial product Atridox^®^ for controlled delivery of doxycycline in periodontitis treatment [57]. Nonetheless, the addition of SAL and MYR did not diminish antimicrobial activity following matrix formation. However, under the present experimental conditions, a distinct enhancement of antimicrobial efficacy attributable solely to SAL or MYR could not be established. Instead, it might lead to a reduction in antimicrobial activity due to the decrease in the amount of NMP by weight. This aligns with our previous findings indicating that the addition of polymers and matrix-forming agents tends to diminish the diameter zone of inhibition owing to the slowing of drug and NMP diffusion from the MYR matrix after ISG exposure to aqueous phase from agar media [21]. Despite this reduction in the inhibition zone following the inclusion of SAL and MYR, the developed ISGs still exhibited antimicrobial effects by producing apparently clear zones against the tested pathogens.

Although direct comparison with free SAL or commercial oral formulations was not performed in this study, the primary objective of the antimicrobial evaluation was to verify that SAL retained its biological activity following incorporation into the MYR-based in situ forming gel system. It should be noted that agar diffusion-based assays are strongly influenced by diffusion rates, which may underestimate the apparent antimicrobial activity of depot-forming systems compared to freely diffusible solutions. In contrast to conventional oral formulations such as rinses or gels, which are rapidly cleared from the oral cavity, the developed ISG is designed to form a localized depot that enables prolonged residence time and the sustained release of SAL at the application site. Previous studies have reported antimicrobial activity of free SAL against oral pathogens [58]; nonetheless, such formulations typically require frequent reapplication due to limited retention. Therefore, even though direct efficacy comparisons were beyond the scope of the present study, the observed antimicrobial activity combined with sustained release behavior suggests potential advantages of the ISG system for localized oral therapy. Further in vivo investigations are needed to establish comparative therapeutic efficacy.

Performance of the salicylic acid loaded MYR-based in situ forming gel was contextualized through comparison with previously reported in situ forming gel and depot-based delivery systems, as summarized in Table 6. The comparison emphasizes parameters relevant to localized drug delivery, including matrix-forming materials, release duration, and reported antibacterial activity.

Table 6 shows that the MYR-based ISG achieved longer sustained release than previously reported fatty acid-based ISGs and maintained antimicrobial activity against oral pathogens. Differences in experimental conditions across studies limit direct comparison, yet the results point to strong potential for prolonged localized oral therapy using the present system.

## 3. Conclusions

The SAL-loaded MYR-based ISG has been successfully formulated in this study. SAL, serving as the model drug, not only exhibited matrix-forming properties due to its low solubility in water but also influenced the physicochemical attributes of the ISG. Higher SAL content altered the ISG’s characteristics by increasing density, viscosity, surface tension, and contact angle. Despite these changes, the developed formulations maintained acceptable spreadability. The inclusion of MYR effectively slowed down the matrix-forming process. Higher MYR content prolonged the matrix formation duration, resulting in a denser gel structure. Despite increased viscosity following MYR addition, the formulations retained acceptable injectability and mechanical properties. There was the successful formation of the SAL-loaded MYR-based ISG and the release of SAL from the matrix, aligning with the topographical SEM analysis of the dry remnants. A comparison between solvents, NMP, and DMSO revealed differences in matrix formation rates. NMP—particularly in combination with MYR—exhibited slower matrix formation, leading to incomplete trapping of SAL in the matrix structure. As a result, sustained release of SAL was less effective compared to ISGs prepared in DMSO, which demonstrated sustained release with drug retention proportional to MYR content, capable of holding the release of SAL for up to 20 days. The release kinetics matched mathematical models, fitting well with the Peppas–Sahlin model dominated by Fickian diffusion, indicating SAL release from the matrix relied on solvent diffusion and chemical concentration gradient.

The developed formulations showed appropriate physicochemical properties, injectability, in situ matrix formation, and sustained salicylic acid release under in vitro conditions. Antimicrobial activity remained after incorporation into the ISG system, although the magnitude of activity varied according to the organic solvent employed. The investigation remains limited to in vitro testing, and further in vivo assessment, cytotoxicity evaluation, and mucosal compatibility analysis are required before therapeutic use can be considered. Overall findings demonstrate the feasibility of a solvent removal-based MYR-SAL in situ forming gel as a proof of concept platform for localized oral drug delivery.

## 4. Materials and Methods

### 4.1. Materials

Myristic acid (MYR) (lot no. FPLK437 × 4S, P.C. drug center Co., Ltd., Bangkok, Thailand) used as gel formation. Salicylic acid (SAL) (lot no. RAS1101900, P.C. drug center Co., Ltd., Bangkok, Thailand) was used as a model drug. NMP (≥99.5%, Lot No. 144560-118, QReC, Auckland, New Zealand), DMSO (≥99.9%, Lot No. 1862992, Fisher Chemical, Leicestershire, UK), and PYR (QReC, Auckland, New Zealand) were used as solvents. *S. aureus* ATCC 25923, *E. coli* ATCC 25922, *S. mutans* ATCC 25175, and *P. gingivalis* ATCC 33277, *C. albicans* TISTR 10231, *C. krusei* TISTR 5259, *C. lusitaniae* TISTR 5156, and *C. tropicalis* TISTR 5306 from Ministry of public health, Nonthaburi District, Thailand were employed as test microbes. The bacterial strains were cultivated on tryptic soy agar (TSA) (lot no. 7341698, Difco, Detroit, MI, USA) and sheep blood agar (Department of Medical Science, Ministry of Public Health, Nonthaburi District, Thailand), while the fungal strains were grown on Sabouraud dextrose agar (SDA) (lot no. 7312647, Difco, Detroit, MI, USA).

### 4.2. Preparation of ISGs

Salicylic acid (SAL) was first dissolved in the selected organic solvent (NMP, DMSO, or PYR) at the specified concentration under continuous magnetic stirring (Benchmark H3770-HS-E Digital Hotplate Stirrer, Benchmark Scientific Inc., Sayreville, NJ, USA) at ambient temperature (25 ± 2 °C) until a clear and homogeneous solution was obtained. Subsequently, myristic acid (MYR) was gradually added to the SAL solution, and stirring was continued until complete dissolution, as confirmed by visual inspection of a transparent, particle-free solution. The formulations were further stirred for 30 min to ensure homogeneity and allowed to stand to remove entrapped air bubbles prior to characterization. No heating was applied during the preparation process to avoid premature crystallization.

As a representative example, formulation SD30M20 was prepared by dissolving 30% *w*/*w* SAL in DMSO followed by gradual addition of 20% *w*/*w* MYR under the same conditions described above until a homogeneous, transparent solution was obtained. The same preparation procedure was applied to all other formulations with appropriate adjustment of component concentrations. All formulations are listed in Table 1 and were freshly prepared and stored in sealed containers at ambient temperature until use.

### 4.3. Physical Property Studies

#### 4.3.1. Viscosities and Density

Viscosity measurements and the shear stress–shear rate relationship were determined using a viscometer (Brookfield Engineering Laboratories Inc., Middleborough, MA, USA) equipped with a CP-40 spindle (n = 3) at 25 °C and a shear rate of 250 s^−1^. The density of each solution was measured using a pycnometer (Mettler-Toledo Ltd., Vernon Hills, IL, USA) (n = 3).

#### 4.3.2. Contact Angle and Surface Tension

Glass and agarose gel surfaces were used to measure contact angles with a drop shape analyzer FTA 1000 from First Ten Angstroms (Portsmouth, VA, USA) as described previously [19,20]. Fluid was dispensed at a pump rate of 1.9 microliters per second, and the contact angle recorded at 5 s after droplet formation with three replicates. Surface tension was determined from changes in the shape of a pendant droplet of the prepared solution suspended in air after injection using the same analyzer and pump rate with three replicates.

#### 4.3.3. Macroscopic Gel Formation Investigation

This direct gel formation process was evaluated by injecting 1 mL of the prepared formulation through stainless-steel needle into phosphate-buffered saline (PBS) at pH 6.8. Gel formation was also examined by introducing 100 µL of the prepared formulation into wells containing agarose dissolved in phosphate-buffered saline at pH 6.8 [37]. Photographic images of the transformation were recorded at 1, 5, 10, 20, and 30 min to monitor the progression of gel formation over time.

#### 4.3.4. Study of Mechanical and Injectability Properties

The mechanical properties of the ISG systems were evaluated using a texture analyzer (TA.XT Plus, Stable Micro Systems Ltd., Surrey, UK). A 0.6% *w*/*w* agarose gel was prepared and molded into wells, each filled with 150 µL of the formulation and left for 3 days to allow complete phase inversion [46]. The analytical probe was then driven into the formed gel at 0.5 mm/s, held for 60 s, and withdrawn at 10 mm/s. The maximum force during penetration represented the maximum deformation force (hardness), while the force upon probe withdrawal indicated the adhesion force. All measurements were performed in triplicate.

Injectability of the ISG formulations was also assessed using the texture analyzer in compression mode [20]. SAL-loaded formulations were filled into 1 mL syringes fitted with 27-gauge needles and fixed to a stainless-steel stand. The probe was driven downward at 1.0 mm/s, applying a 0.1 N force to the syringe plunger. The area under the force–distance curve was recorded as the work of injection (N·mm). Experiments were conducted in triplicate.

#### 4.3.5. In Vitro Drug Release Studies

The in vitro drug release study for ISG formulations was investigated using a porcine cup method. First, 0.3 g of SAL-loaded MYR-based ISG formulations and control formulations (SAL-organic solvent) were filled into cup, then filled cup was put into 80 mL PBS pH 6.8, shaking was maintained at 37 °C with gentle agitation at 50 rpm [40]. At predetermined time intervals, 3 mL of the release medium was withdrawn and replaced with an equal volume of fresh PBS to preserve sink conditions. The concentration of SAL in the collected samples (n = 6) was quantified using a UV–VIS spectrophotometer (Agilent Cary 60 UV–VIS Spectrophotometer, Santa Clara, CA, USA) at a wavelength of 295 nm. A calibration curve was constructed using SAL standard prepared in the same release medium.

The drug release mechanisms were analyzed by fitting the dissolution data to various mathematical models, including zero-order, first-order, Higuchi, Korsmeyer–Peppas, and Peppas–Sahlin models. The DD-Solver software, an add-in program for Microsoft Excel (Redmond, WA, USA) developed using Visual Basic for Applications, was employed to determine the most suitable release model. The n-value obtained from the Korsmeyer–Peppas equation was used to elucidate the predominant drug release mechanism.

#### 4.3.6. Scanning Electron Microscopy (SEM)

Following the drug release experiment in PBS (pH 7.4), the remaining gel was rinsed with 200 mL of distilled water and subsequently freeze-dried. The dried samples were stored in a desiccator for 72 h and coated with a thin layer of gold using a sputter coater (BIO-RAD SEM Coating Unit PS3, BIO-RAD Laboratories Ltd., Hercules, CA, USA) prior to analysis. The surface morphology of the ISG remnants was examined using a scanning electron microscope (SEM) (TESCAN MIRA3, Brno-Kohoutovice, Czech Republic) operated at an accelerating voltage of 15 kV.

### 4.4. Antimicrobial Activity

Antimicrobial activities were undertaken using the agar cup diffusion method under the approval of the Walailak University Institutional Biosafety Committee (No. WU-IBC-68-058) [44,46]. The test formulations comprisedthe prepared ISGs and control groups while the microbes included the standard bacteria (*S. aureus* ATCC 6538P and *E. coli* ATCC 25922), anaerobic pathogen microbe (*P. gingivalis* ATCC 33277), and fungal species (*C. tropicalis* TISTR 5306, *C. albicans* ATCC 17110, *C. lusitaniae* TISTR 5156 and *C. krusei* TISTR 5259). Standard bacteria, anaerobic pathogen, and fungal inocula, adjusted to approximately 0.5 McFarland turbidity, were spread on tryptic soy agar, sheep blood agar, and Sabouraud dextrose agar, respectively. Sterilized cylindrical cups were placed on the agar surface, and 200 μL of the prepared ISG or ISM was added to each cup. Plates were incubated at 37 °C for 24 h, while anaerobic bacteria were incubated at 37 °C for 72 h in an anaerobic incubator (Forma Anaerobic System, Thermo Scientific, Bedford, OH, USA). Antimicrobial activity was evaluated by measuring the diameter of the inhibition zone (mm), with experiments performed in triplicate (n = 3).

### 4.5. Statistical Analysis

All data were analyzed using one-way analysis of variance (ANOVA) followed by Tukey’s post hoc test. Differences were considered statistically significant at *p* < 0.05. Statistical analyses were performed using SPSS for Windows (version 11.5).

## Figures and Tables

**Table 1:** Composition of SAL-loaded MYR-based ISG formulations.

Formulation Code *	SAL (% *w*/*w*)	MYR (% *w*/*w*)	Organic Solvent (Adjust to 100% *w*/*w*)
SAL-only formulations (without MYR)			
SN10	10	-	NMP
SN20	20	-	NMP
SN30	30	-	NMP
SN40	40	-	NMP
SN50	50	-	NMP
SN60	60	-	NMP
SD10	10	-	DMSO
SD20	20	-	DMSO
SD30	30	-	DMSO
SD40	40	-	DMSO
SD50	50	-	DMSO
SD60	60	-	DMSO
SPy10	10	-	PYR
SPy20	20	-	PYR
SPy30	30	-	PYR
SPy40	40	-	PYR
SAL + MYR formulations			
SN30M25	30	25	NMP
SD30M20	30	20	DMSO
SD30M25	30	25	DMSO
SD30M30	30	30	DMSO

* SN, SD, and SPy denote formulations prepared using NMP, DMSO, and PYR as solvents, respectively. The number indicates SAL concentration (% *w*/*w*). Formulations containing “M” indicate the presence of MYR, with the number following “M” representing MYR concentration (% *w*/*w*).

**Table 2:** Physical properties of SAL in organic solvent and SAL-loaded solvent removal MYR-based ISG systems (n = 3).

Formula	Density ± S.D. (g/cm^3^)	Viscosity ± S.D. (cP)	Surface Tension ± S.D. (mN/m)	Contact Angle ± S.D. (Degree)	
				Glass Slide	Agarose Gel
SN10	1.0548 ± 0.0000	3.37 ± 0.01	41.00 ± 0.38	15.30 ± 0.72	6.63 ± 0.68
SN20	1.0774 ± 0.0001	3.60 ± 0.02	41.33 ± 0.22	21.32 ± 1.04	8.54 ± 0.19
SN30	1.1006 ± 0.0002	5.20 ± 0.01	41.83 ± 0.07	27.95 ± 1.62	11.45 ± 0.61
SN40	1.1247 ± 0.0002	7.89 ± 0.01	42.36 ± 0.67	30.69 ± 1.23	18.24 ± 0.37
SN50	1.1503 ± 0.0001	14.06 ± 0.06	43.05 ± 0.50	30.94 ± 0.90	23.43 ± 0.56
SN60	1.1755 ± 0.0003	28.80 ± 0.32	43.45 ± 0.36	32.25 ± 1.75	26.84 ± 1.91
SD10	1.1154 ± 0.0001	3.25 ± 0.08	43.69 ± 0.09	16.52 ± 2.13	7.48 ± 0.22
SD20	1.1288 ± 0.0001	3.93 ± 0.02	43.70 ± 0.05	17.46 ± 1.91	8.77 ± 0.27
SD30	1.1464 ± 0.0001	4.98 ± 0.01	43.94 ± 0.28	22.95 ± 0.83	12.41 ± 0.26
SD40	1.1641 ± 0.0002	6.76 ± 0.01	43.39 ± 0.24	24.91 ± 3.49	13.06 ± 1.76
SD50	1.1840 ± 0.0001	10.04 ± 0.02	43.69 ± 0.23	26.18 ± 2.69	16.32 ± 0.82
SD60	1.2043 ± 0.0002	16.06 ± 0.03	43.43 ± 1.22	31.44 ± 0.85	22.23 ± 1.11
SPy10	1.1254 ± 0.0002	19.61 ± 0.02	52.39 ± 0.10	19.71 ± 1.11	6.00 ± 0.02
SPy20	1.1414 ± 0.0001	24.80 ± 0.01	63.32 ± 0.22	22.35 ± 1.12	6.35 ± 0.28
SPy30	1.1580 ± 0.0002	35.78 ± 0.01	64.52 ± 0.34	32.52 ± 0.06	12.34 ± 0.28
SPy40	1.1735 ± 0.0002	48.28 ± 0.32	78.31 ± 0.36	34.63 ± 0.18	15.96 ± 1.51
SN30M25	1.0542 ± 0.0001	13.45 ± 0.03	34.54 ± 0.08	20.76 ± 0.70	26.20 ± 0.25
SD30M20	1.0911 ± 0.0001	9.59 ± 0.02	32.72 ± 0.37	19.68 ± 1.51	20.24 ± 0.78
SD30M25	1.0794 ± 0.0000	11.82 ± 0.04	31.95 ± 0.05	19.00 ± 0.75	29.16 ± 0.74
SD30M30	1.0688 ± 0.0001	14.43 ± 0.01	31.41 ± 0.16	18.38 ± 0.66	30.92 ± 0.12
NMP	1.0265 ± 0.0008	2.04 ± 0.13	39.31 ± 0.28	31.08 ± 0.40	7.12 ± 1.51
DMSO	1.0935 ± 0.0007	1.98 ± 0.09	43.95 ± 0.13	33.98 ± 1.25	4.30 ± 1.19
PYR	1.1071 ± 0.0006	11.46 ± 0.19	42.77 ± 1.03	44.16 ± 1.42	6.01 ± 0.33

**Table 3:** Injectability and mechanical properties of SAL-loaded MYR-based ISGs and pure organic solvent (n = 3).

Formulation	Injectability Properties		Maximum Force ± S.D. (N)	Remaining Force ± S.D. (N)	Adhesion Force ± S.D. (N)	Mechanical Properties ± S.D.
	Injection Force ± S.D. (N)	AUC of Injection ± S.D. (N∙mm)				
DMSO	0.734 ± 0.047	12.339 ± 0.375	ND	ND	ND	ND
SD30	1.274 ± 0.056	23.492 ± 0.364	0.203 ± 0.053	0.057 ± 0.023	0.093 ± 0.016	0.275 ± 0.043
SD30M20	2.032 ± 0.030	36.784 ± 0.068	0.651 ± 0.070	0.172 ± 0.022	0.087 ± 0.011	0.264 ± 0.013
SD30M25	2.475 ± 0.114	44.357 ± 3.576	1.164 ± 0.182	0.164 ± 0.040	0.096 ± 0.007	0.139 ± 0.014
SD30M30	2.805 ± 0.120	50.745 ± 1.133	1.760 ± 0.179	0.564 ± 0.016	0.236 ± 0.007	0.323 ± 0.038
NMP	0.904 ± 0.171	12.195 ± 0.369	ND	ND	ND	ND
SN30	1.125 ± 0.018	21.216 ± 0.423	0.478 ± 0.080	0.058 ± 0.003	0.087 ± 0.012	0.123 ± 0.024
SN30M25	2.739 ± 0.022	51.221 ± 0.633	1.130 ± 0.076	0.307 ± 0.024	0.095 ± 0.005	0.272 ± 0.013

**Table 4:** Degree of goodness-of-fit and estimate parameters from curve fittings of the SAL release profiles of different ISMs in PBS pH 6.8 using cup method (n = 3).

Formulation	Modeling	Parameters	R^2^	AIC	MSC
SD30	Zero order	k_0_ = 5.67	0.3183	139.4282	0.2699
	First order	k_1_ = 0.125	0.8013	199.1436	1.5376
	Higuchi’s	k_H_ = 20.392	0.9289	102.1295	2.6010
	Korsmeyer–Peppas	k_KP_ = 27.505	0.9876	74.8106	4.3085
		n = 0.361			
	Peppas–Sahlin	k_1_ = 25.274	0.9890	72.6945	4.4407
		k_2_ = 1.96			
		m = 0.367			
SD30M25	Zero order	k_0_ = 3.755	0.5138	135.912	0.6427
	First order	k_1_ = 0.062	0.7743	122.8604	1.4104
	Higuchi’s	k_H_ = 14.102	0.9523	92.2911	3.2086
	Korsmeyer–Peppas	k_KP_ = 17.568	0.9775	82.8940	3.7614
		n = 0.409			
	Peppas-Sahlin	k_1_ = 17.701	0.9931	65.0956	4.8084
		k_2_ = −1.416			
		m = 0.573			
SN30	Zero order	k_0_ = 10.914	0.3830	123.295	0.3486
	First order	k_1_ = 0.275	0.8074	107.0197	1.5112
	Higuchi’s	k_H_ = 30.213	0.9130	95.5640	2.3295
	Korsmeyer-Peppas	k_KP_ = 39.739	0.9872	69.9562	4.1586
		n = 0.341			
	Peppas–Sahlin	k_1_ = 39.984	0.9866	71.3048	4.0623
		k_2_ = −0.165			
		m = 0.348			
SN30M25	Zero order	k_0_ = 10.925	0.4483	123.2305	0.5588
	First order	k_1_ = 0.294	0.8489	102.7745	2.0199
	Higuchi’s	k_H_ = 30.258	0.9015	96.7123	2.4529
	Korsmeyer–Peppas	k_KP_ = 37.499	0.9607	87.9903	3.0759
		n = 0.382			
	Peppas–Sahlin	k_1_ = 44.285	0.9764	81.9331	3.5086
		k_2_ = −6.085			
		m = 0.539			

**Table 5:** Clear zone diameter of SAL-loaded MYR-based ISG systems against *S. aureus*, *E. coli*, *P. gingivalis*, *C. albicans*, *C. krusei*, *C. lusitaniae* and *C. tropicalis* (n = 3).

Formula	Clear Zone Diameter (mm) Mean ± S.D.)						
	*S. aureus* 6538	*E. coli* 8739	*P. gingivalis* 33277	*C. albicans* 10231	*C. krusei* 5259	*C. lusitaniae* 5156	*C. tropcalis* 5306
NMP	17.0 ± 1.0 ^a^	17.7 ± 1.5	17.0 ± 1.0 ^f^	28.0 ± 2.0 ^h^	23.7 ± 1.2 ^j^	28.3 ± 1.5	25.8 ± 0.8 ^m^
SN30	20.3 ± 0.6	15.0 ± 1.0	21.3 ± 0.6	31.1 ± 1.2 ^h^	27.0 ± 1.0 ^j^	31.7 ± 1.5	30.3 ± 0.6 ^m^
SN30M25	17.5 ± 0.6 ^a^	12.2 ± 0.3	18.8 ± 1.6 ^f^	35.3 ± 2.3	24.3 ± 2.5 ^j^	35.0 ± 1.7	30.3 ± 4.9 ^m^
DMSO	12.2 ± 1.0 ^b^	12.0 ± 0.6 ^d^	14.3 ± 0.6 ^g^	16.3 ± 0.6	15.8 ± 1.3 ^k^	20.8 ± 0.8 ^l^	19.8 ± 1.3 ^n^
SD30	20.7 ± 0.6	18.7 ± 0.6	17.3 ± 2.5 ^g^	30.7 ± 0.6	25.7 ± 1.5	31.3 ± 0.6	23.7 ± 1.5 ^n^
SD30M20	14.3 ± 0.6 ^c^	11.2 ± 0.3 ^d,e^	17.2 ± 0.3 ^g^	20.0 ± 1.0 ^i^	21.0 ± 1.0	24.7 ± 0.6	20.3 ± 0.6 ^n^
SD30M25	14.5 ± 0.5 ^c^	10.7 ± 0.6 ^d,e^	16.8 ± 0.3 ^g^	20.3 ± 0.6 ^i^	20.0 ± 0.0	24.2 ± 0.3	20.0 ± 0.0 ^n^
SD30M30	12.7 ± 0.8 ^b,c^	9.3 ± 0.6 ^e^	14.0 ± 0.5 ^g^	20.3 ± 0.6 ^i^	17.7 ± 0.6 ^k^	22.7 ± 0.6 ^l^	19.0 ± 1.0 ^n^

The superscripts ^a–n^ indicate a no significant difference (*p* < 0.05) by using one-way ANOVA followed by an LSD post hoc test.

**Table 6:** Comparison of the present ISG system with previously reported in situ forming gel formulations.

Study	Drug	Matrix Former	Solvent/System	Release Duration	Antibacterial Activity
Ref. [18]	Vancomycin	Lauric acid	Solvent removal ISG	~6 days	Yes (oral pathogens)
Ref. [48]	Etoposide	poly(lactic-co-glycolic acid)	ISG	~30 days	Not applicable
Ref. [21]	Metronidazole	Camphor	NMP-based	~7 days	Yes (oral pathogens)
This study	Salicylic acid	Myristic acid	DMSO-based ISG	Up to 20 days	Yes (oral pathogens)

## Data Availability

Data is contained within the article and available upon request.
